# The Mitochondrial tRNA^Phe^ 625G>A Mutation in Three Han Chinese Families With Cholecystolithiasis

**DOI:** 10.3389/fgene.2022.814729

**Published:** 2022-05-27

**Authors:** Lingling Hou, Cuifang Hu, Lili Ji, Qiongdan Wang, Min Liang

**Affiliations:** ^1^ Department of Medical Laboratory, The First Affiliated Hospital of Wenzhou Medical University, Wenzhou, China; ^2^ Attardi Institute of Mitochondrial Biomedicine, Wenzhou Medical University, Wenzhou, China

**Keywords:** cholecystolithiasis, mitochondrial, tRNA, mutation, pedigree

## Abstract

In this study, we assessed three Chinese families with inherited cholecystolithiasis and conducted the clinical, genetic, and molecular characterization of these subjects. Eight of eighteen matrilineal relatives had a clinical phenotype in these three families. Sequence analysis of complete mitochondrial genomes in these probands identified the homoplasmic tRNA^Phe^ 625 G > A mutation and distinct sets of mtDNA polymorphisms belonging to haplogroups H2, F4b, and M10a. The 625G > A mutation disturbed the classic G-C base-pairings at a highly conserved position 49 in the T-stem of mitochondrial tRNAs. Molecular dynamics simulation showed that the structure of tRNA^phe^ with 625 G > A mutation was noticeably remodeled while compared with the isoform of the wild type. The occurrence of tRNA^Phe^ 625 G > A mutation in these various genetically unrelated subjects strongly indicates that this mutation is involved in the pathogenesis of cholecystolithiasis. This is the first evidence that tRNA mutations are associated with cholecystolithiasis, and it provided more insights into the genetic mechanism of cholecystolithiasis.

## Introduction

Cholecystolithiasis and its associated complications (e.g., cholecystitis and pancreatitis) have become an increasingly significant public health problem around the globe. ∼20%–30% of adults in developed countries have been diagnosed with cholecystolithiasis (Rebholz et al., 2018). The etiology of cholecystolithiasis is complicated, and age, sex, pregnancy, alcohol, coffee, and other factors are usually involved (Morimoto et al., 2021). In particular, cholecystolithiasis has a strong genetic predisposition, and ethnic differences indicate that genetic factors play an important role in the occurrence of cholecystolithiasis (Gilat et al., 1983; Lammert et al., 2005; Wittenburg et al., 2007; Buch et al., 2010). So far, several nuclear genes have been identified in cholecystolithiasis patients (Buch et al., 2010). However, the association between mitochondrial DNA (mtDNA) and the onset of cholecystolithiasis still remains to be unraveled. The mitochondrion provides energy for various physiological activities of eukaryotic cells and plays an important role in the metabolism of glucose and lipids (Mahmood et al., 2016; García-Berumen et al., 2019). So, mutations in mtDNA can cause many metabolic diseases, including mitochondrial myopathy, mitochondrial encephalomyopathy, hypertension, type 2 diabetes, and dyslipidemia (Goto, 2000; Rahman, 2012; Xue et al., 2016; Rovira-Llopis et al., 2017; Rumora et al., 2018; De Barcelos et al., 2019; Pinti et al., 2019; Radelfahr and Klopstock, 2019; Zhao et al., 2019; Prasun, 2020).To further elucidate the molecular basis of cholecystolithiasis in the Chinese population, a systematic and extended mutational screening of mtDNA has been initiated in a large clinical population of the First Affiliated Hospital of the Wenzhou Medical College (Hou et al., 2018). In the current study, we performed the clinical, genetic, and molecular characterizations of three Chinese families with inherited cholecystolithiasis. Molecular analysis identified the homoplasmic 625G > A mutation, which localized at a highly conserved position 49 in the T-stem in mitochondrial tRNA^Phe^. To elucidate the role of mitochondrial haplotype in the phenotypic manifestation of the 625G > A mutation, we conducted high-throughput sequencing and Sanger sequencing spanning the entire mitochondrial genome and subsequent DNA sequence analysis of three probands in those Chinese families. Furthermore, we computed the structural analysis and simulations of molecular dynamics of the mitochondrial tRNA gene.

## Materials and Methods

### Patients

**FIGURE 1:**
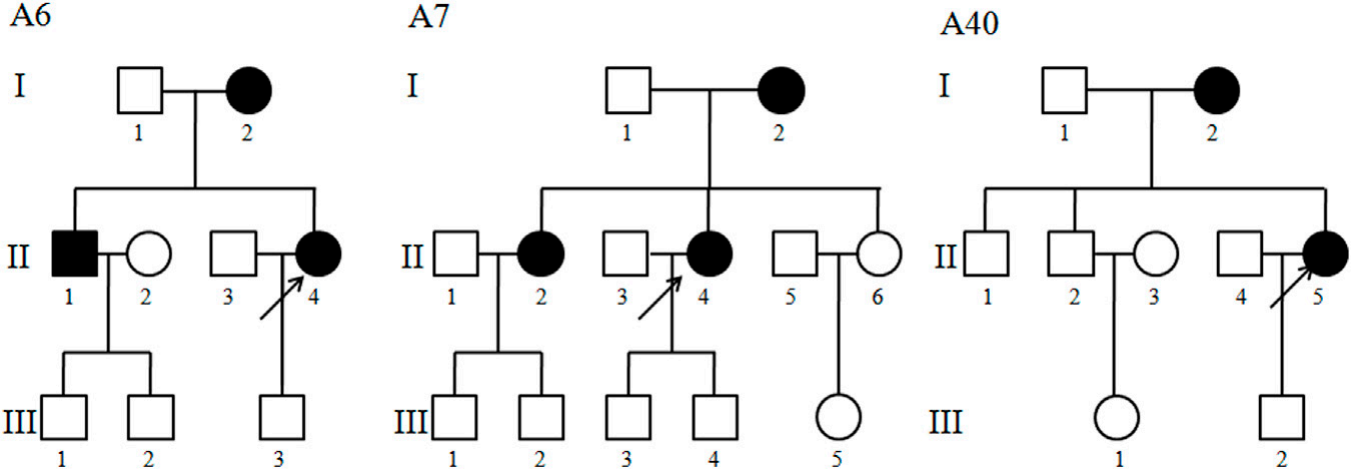
Three Han Chinese pedigrees with cholecystolithiasis. Cholecystolithiasis individuals are indicated by filled symbols. An arrow denotes proband.

Three Chinese cholecystolithiasis families, as shown in Figure1, were ascertained through the First Affiliated Hospital of Wenzhou Medical University. Informed consent, blood samples, and clinical evaluations were obtained from all participating family members, under protocols approved by the Wenzhou Medical University ethics committee. A comprehensive history, blood biochemistry, imaging examination, and long-term follow-up for these participating subjects were conducted at length to identify both personal or family medical histories of individual cholecystolithiasis disease and other clinical abnormalities.

### Mutational Analysis of the Mitochondrial Genome

DNA was isolated from the whole blood of participants by using the DNA extraction reagent (TaKaRa Universal Genomic DNA Extraction Kit Ver3.0) The cholecystolithiasis group DNA samples and the 300 Chinese control samples with concentrations over 87.5 ng/μl were sent to Suichuan Biological Co., Ltd. for VariantPro^*TM*^ Amplicon experiments after evaluation and quality check. This study used the Illumina HisSeq2500 as a high-throughput sequencing method for sequencing. The system uses two patented technologies: OmegaPrimer^*TM*^ and RelayPCR^*TM*^ to conduct the entire process from target sequence capture to sequencing library preparation in one step, which has good library uniformity, repeatability, sensitivity, and specificity. To further verify the mutations of the high-throughput sequencing, the entire mitochondrial genome of three probands was PCR amplified in 24 overlapping fragments using sets of the light (L) strand and the heavy (H) strand oligonucleotide primers as described previously (Liang et al., 2009; Zhao et al., 2009). Each fragment was purified and subsequently analyzed by direct sequencing in an ABI 3700 automated DNA sequencer using the Big Dye Terminator Cycle (Applied Biosystems, Foster City, CA) sequencing reaction kit. These sequence results were compared with the updated consensus Cambridge sequence (GenBank accession number: NC_012920) (Brandon et al., 2005). These variants were evaluated for the pathogenicity using the following criteria: 1) present in <1% of the controls; 2) conservation indexes (CI) > 75%; 3) potential structural and functional alterations; and 4)MitoTip Scores >50%(Liang et al., 2009; Zhao et al., 2009).

### Haplogroup Analysis

The Asian mitochondrial haplogroup of these three probands was determined using online software (www.mitotool.org/genomeRSRS.html) or based on the nomenclature of mitochondrial haplogroups previously reported (Tanaka et al., 2004; Kong et al., 2006).

### Structural Analysis

The published secondary structures for the tRNAs were used to define the stem and loop structure (Florentz et al., 2003; Ruiz-Pesini and Wallace, 2006; Suzuki and Nagao, 2011). The tRNA sequences and secondary structures of corresponding species were downloaded from the relevant database (http://trna.bioinf.uni-leipzig.de/DataOutput/Search); then, the FASTA format of the sequence was submitted to web servers for RNA secondary structure prediction (http://rna.urmc.rochester.edu/RNAstructureWeb).

### Phylogenetic Analysis

A total of 17 vertebrate mitochondrial DNA sequences from the NCBI database were used in the interspecific analysis. The conservation index (CI) was calculated by comparing the human nucleotide variants with other 16 vertebrates. The CI was then defined as the percentage of species from the list of 17 different vertebrates that have the wild-type nucleotide at that position (Liang et al., 2014).

## Result

### Clinical Presentation

The proband (II-4) with a gallstone in family A6 was found 7 years ago, she suffered persistent pain in the right upper quadrant for 1 month, 1 month later, her back suffered radiation pain, with the help of ultrasound, she was diagnosed as cholecystolithiasis with chronic cholecystitis. As shown in Figure 1 and Table 1, 3 of 4 matrilineal relatives had a history of cholecystolithiasis, and the penetrances of cholecystolithiasis in this family were 75%. In family A7, the proband (II-4) was 42 years old, gallbladder stones were found during a routine physical examination 4 years ago, and now she seeks medical care at the First Affiliated Hospital of Wenzhou Medical University because of the unbearable pain caused by gallstones. In fact, 3 of 9 matrilineal relatives had a history of cholecystolithiasis, and the penetrances in this family were 33.3%. The proband (II-5) of family A40 was 43 years old, suffered paroxysmal cramps in the right upper abdomen for 5 days, and after B type ultrasonic examination and combined with clinical manifestations, and then she was diagnosed with cholecystolithiasis. Interestingly, her mother also had a history of cholecystolithiasis.

**TABLE 1:** Summary of the clinical data for eighteen maternal relatives in three Han Chinese cholecystolithiasis pedigrees.

Subjects	Sex	Age at	Age at	Weight	Height	BMI	Glu	Bp	TC	TG	HDL	LDL
		Test (year)	Onset (year)	(Kg)	(cm)	(Kg/m^2^)	(mmol/L)	(mmHg)	(mmol/L)	(mmol/)	(mmol/)	(mmol/)
A6-I-2	Female	60	42	62	154	26.14	4.2	130/79	4.91	1.22	1.42	2.84
A6-II-1	Male	41	39	70	172	23.66	4.0	112/78	4.55	1.42	1.36	2.97
A6-II-4	Female	38	31	60	155	24.97	4.9	116/76	4.99	1.63	1.06↓	3.28
A6-III-3	Male	14		38	150	16.89	3.9	112/85	5.02	1.44	1.33	2.62
A7-I-2	Female	65	46	60	156	24.65	4.2	129/80	4.67	1.38	1.38	3.31
A7-II-2	Female	43	40	63	157	25.56	3.8	122/78	4.42	1.62	1.32	3.02
A7-II-4	Female	42	38	67	160	26.17	4.4	126/71	3.89	1.02	1.28↓	2.31
A7-II-6	Female	38		55	154	23.19	4.1	118/70	4.12	1.12	1.40	2.78
A7-III-1	Male	21		70	171	23.94	3.6	118/80	4.24	1.24	1.42	2.97
A7-III-2	Male	19		68	169	23.81	4.2	119/80	4.36	1.34	1.42	3.02
A7-III-3	Male	21		64	169	22.41	3.9	108/78	3.88	1.42	1.38	2.66
A7-III-4	Male	16		65	160	25.39	3.5	108/76	4.12	1.51	1.34	2.65
A7-III-5	Female	6		24	115	18.15	-	116/78	-	-	-	-
A40-I-2	Female	66	31	60	165	22.04	3.7	127/82	4.12	1.52	1.42	3.3
A40-II-1	Male	46		75	171	25.65	4.2	119/78	4.36	1.32	1.34	2.78
A40-II-2	Male	44		74	170	25.61	3.9	118/70	5.08	1.33	1.38	2.56
A40-II-5	Female	43	43	80	162	30.48↑	6.0	130/86↑	5.52	1.55	0.97↓	3.62↑
A40-III-2	Male	20		68	170	23.53	4	116/80	5.12	1.23	1.42	3.02

BMI, body mass index; Bp, blood pressure; Glu, blood glucose; TC, total cholesterol; TG, triglyceride; HDL, high-density lipoprotein; LDL, low-density lipoprotein;: ↓decrease; ↑: increase.

As shown in Table 1, 8 (1 male and 7 female) of 18 matrilineal relatives had a clinical phenotype in these three families, with an average age of onset was 38.7 years. The proband of the A40 family (II-5) weighed 80 kg and had a BMI of 30.48, which indicated that it may be an overweight patient. Blood pressure monitoring showed that the proband of the A6 and A7 family (II-4, II-4) had normal blood pressure, but the proband of the A40 family (II-5) had systolic pressure slightly higher than normal, which was at the critical level. Blood biochemical analysis showed that the blood glucose levels, total cholesterol, and triglyceride concentrations of the three probands were within the normal range, that is the normal values were 3.89–6.11 mmol/L, 2.8–5.17 mmol/L, and 0.56–1.7 mmol/L. The High-Density Lipoprotein (HDL) levels decreased obviously in all three probands, especially A40-II-5, which measured only 0.97 mmol/L, significantly lower than the normal level of 1.29–1.55 mmol/L. Moreover, the Low-Density Lipoprotein (LDH) was measured at 3.62 mmol/L in A40-II-5, higher than the normal 2.07–3.37 mmol/L, while the other two probands have a normal level. The other five patients in these three families have normal blood glucose levels, total cholesterol, and triglyceride concentrations. Furthermore, there is no evidence that any member of those families had any other known cause to account for cholecystolithiasis. Comprehensive family medical histories of these individuals showed no other clinical abnormalities, including diabetes, muscular diseases, hearing impairment, and neurological disorders.

### Mitochondrial DNA Analysis

**FIGURE 2:**
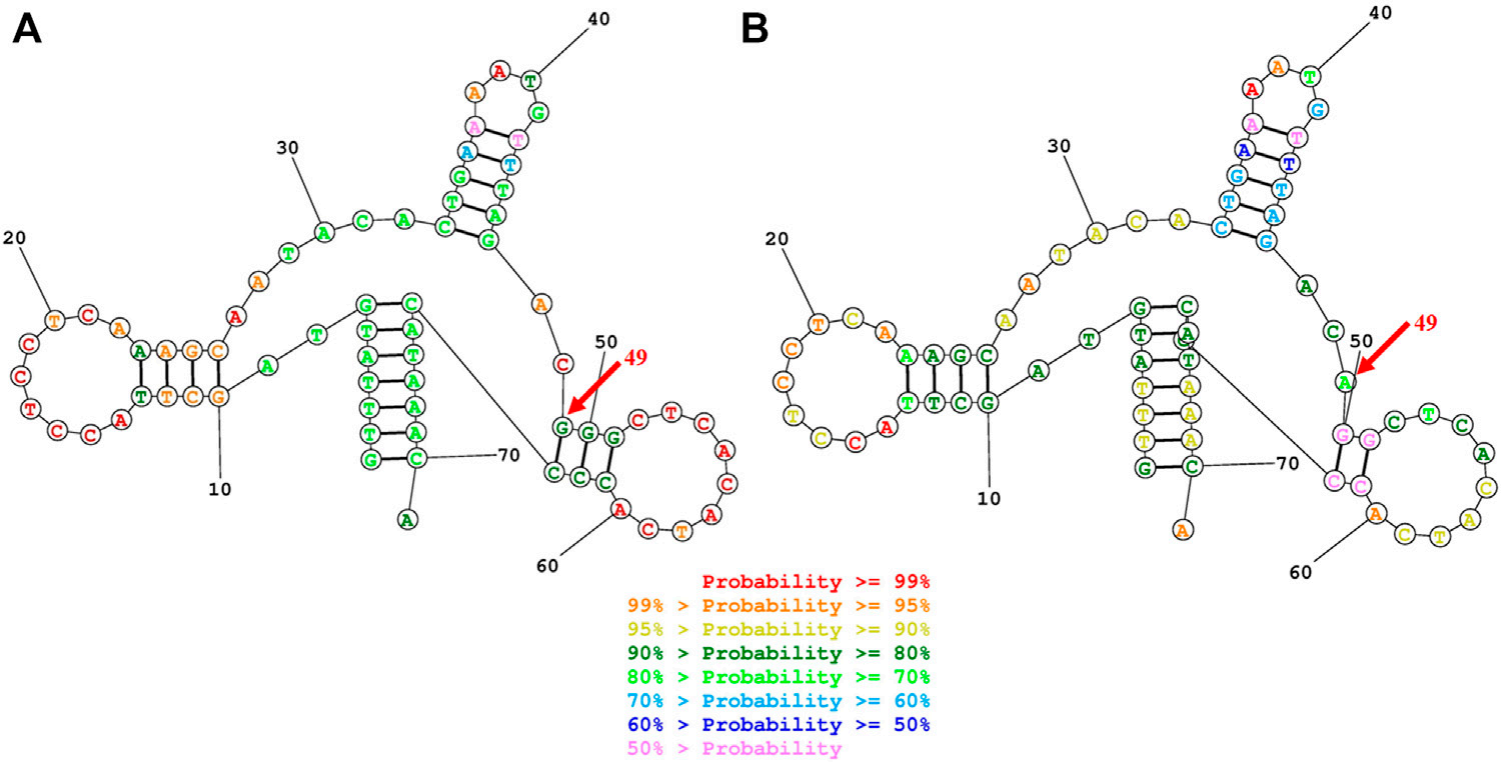
Secondary structure prediction of tRNA^Phe^ by the Web Servers for RNA Secondary Structure Prediction (http://rna.urmc.rochester.edu/RNAstructureWeb). **(A)** G49 residue formed a G-C base pairings in the T-stem of the mitochondrial tRNA^Phe^gene **(B)**The 625G > A mutation damaged the G-C Base pairing at the 49 residues and leads to a disruption of the secondary structure of the tRNA after the loss of the T-stem.

**FIGURE 3:**
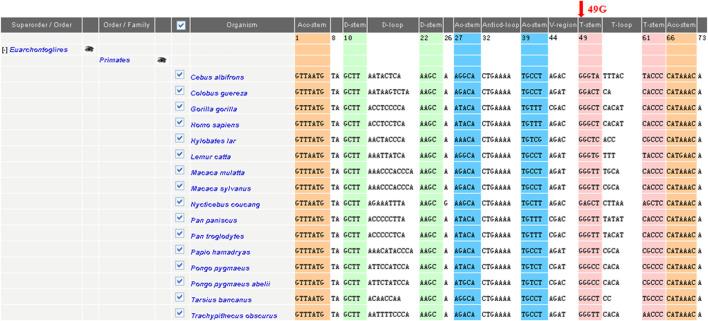
Sequence alignment of the mitochondrial tRNA^Phe^ gene in different species. Arrow indicates the conservation of the G nucleotide at position 49 throughout species. The conservation index (CI) at position 49was 100% in 17 vertebrates, by comparing the human nucleotide variants with other 16 vertebrates.

To elucidate the molecular basis of cholecystolithiasis, we have performed a mutational analysis of the mitochondrial genome in these families. First, PCR amplification of the mitochondrial tRNA gene revealed three families carrying the m.625G > A mutation, which was not present in normal controls. As shown in Figure 2, the homoplasmic m.625G > A mutation affects the G49 residue in the T-stem of the mitochondrial tRNA^Phe^ gene and leads to a disruption of the secondary structure of the tRNA after the loss of the T-stem. This modification could also affect the tertiary structure of the tRNA^Phe^ and had probably a consequence on the aminoacylation. Guanine at this position is a conserved base in sequenced threonine tRNA from bacteria to human mitochondria (Ruiz-Pesini and Wallace, 2006). As shown in Figure 3, among 17 organisms, the Conservative index (CI) of this gene at position 49 in tRNA^Phe^ is 100%. Indeed, MitoTIP Sub-Scoring for variant 625G > A is 81.30%, and it is more likely pathogenic (https://www.mitomap.org/mitomaster/index_snvs.cgi).

We then conducted high-throughput sequencing and Sanger sequencing spanning the entire mitochondrial genome and subsequent DNA sequence analysis of three probands in those Chinese families. In addition to the identical m.625G > A mutation, as shown in Table 2, these subjects exhibited distinct sets of mtDNA polymorphisms, including eighteen known variants in the D-loop, five known variants, and one unknown variant in the 12S rRNA gene, thirty-four known silent variants in the protein-encoding genes, and fourteen known missense mutations in the protein-encoding genes. These missense mutations are the 5263C > T (A265V) in the *ND2* gene, the 8603T > C (F26S), 8701A > G (T59A), 8821T > G (S99A), and 8860A > G (T112A) in *ATPase6* gene, the 9966G > A (V254T) in the *COIII* gene, the 10398A > G (T114A) and 10400C > T (T114A)in the *ND3* gene, the 13135G > A (A267T) and 13928G > C (S531T) in the *ND5* gene, the 14766C > T (T7I),15071T > C(Y109H), 15218A > G (T158A), and 15326A > G (T194A) in the *Cytb* gene. These variants in RNAs and polypeptides were further evaluated by phylogenetic analysis of these variants and sequences from other 16 organisms (Liang et al., 2014). However, all these variants showed no evolutionary conservation. Based on the nomenclature of mitochondrial haplogroups (Tanaka et al., 2004; Kong et al., 2006), we used the mtDNA sequence variations among 3 Chinese probands to establish the haplogroup affiliation of each mtDNA. Here, mtDNAs of pedigree A6, A7, and A40 belong to the Eastern Asian halpogroups H2, F4b, and M10a, respectively. To further exclude the influence of nuclear genes, we conducted a whole exon sequencing of the three probands, but we did not find the nuclear susceptibly genes (dates were not shown).

**TABLE 2:** mtDNA variants in three Chinese pedigrees with cholecystolithiasis.

Gene	Position	Replacement	AA change	CI (%)	A6	A7	A40
*D-loop*	73	A-G			G	G	G
	146	T-C					C
	263	A-G			G	G	G
	310	T-C			C	C	C
	489	T-C					C
	16093	T-C					C
	16129	G-A					A
	16172	T-C				C	
	16182	A-C			C		
	16183	A-C			C		
	16189	T-C			C		
	16218	C-T				T	
	16223	C-T					T
	16261	C-T			T		
	16264	C-T				T	
	16304	T-C				C	
	16311	T-C				C	C
	16519	T-C			C	C	
*tRNA* ^*Phe*^	592	C-T		31.25	T	T	T
	596	T-C		50		C	
	621	A-C		68.75	C	C	C
	625	**G-A**		**100**	**A**	**A**	**A**
	632	C-T		71.43	T	T	T
	634	T-C		66.67	C	C	C
*12S rRNA*	661	C-G			G	G	G
	686	A-G				G	
	708	C-T			T	T	T
	709	G-A					A
	750	A-G			G	G	G
	1438	A-G			G	G	G
*ND1*	3970	C-T				T	
	4140	C-T					T
	4769	A-G			G	G	G
*ND2*	5263	C-T	p.A265V	35.29		T	
	5465	T-C			C		
*ND2*	5814	T-C		88.23	C		
*tRNA* ^*Cys*^	6357	C-T					T
*COI*	6392	T-C				C	
	6653	C-T				T	
	7025	A-G					T
	7028	C-T			T	T	T
	7741	T-C				C	
*COII*	8020	G-A				A	
	8575	C-T				T	
*ATPase6*	8603	T-C	p.F26S	82.35		C	
	8701	A-G	p.T59A	52.94			G
	8793	T-C					C
	8856	G-A					A
	8860	A-G	p.T112A	70.59	G	G	G
	9123	G-A			A		
	9266	G-A				A	
	9540	T-C					C
*COIII*	9764	C-T				T	
	9966	G-A	p.V254I	70.59			A
	10310	G-A				A	
	10398	A-G	p.T114A	41.18			G
*ND3*	10400	C-T	p.T114A				T
	10646	G-A					A
	10873	T-C					C
*ND4L*	11719	G-A			A	A	A
*ND4*	12007	G-A				A	
	12549	C-T					T
	12630	G-A				A	
*ND5*	12705	C-T					T
	12732	T-C			C		
	12879	T-C				C	
	13135	G-A	p.A267T	23.53			A
	13152	A-G					G
	13928	G-C	p.S531T	11.76		C	
	14502	T-C	p.I58V	76.47			C
	14518	A-G			G		
*ND6*	14766	C-T	p.T7I	47.06	T	T	T
	14783	T-C					C
*Cytb*	15040	C-T					T
	15043	G-A					A
	15071	T-C	p.Y109H	29.41			C
	15218	A-G	p.T158A	76.47			G
	15301	G-A					A
	15326	A-G	p.T194A	52.94	G	G	G
	15670	T-C				C	

## Disccusion

Cholecystolithiasis is very common in the general population and is an important cause of hospitalization (Marschall and Einarsson, 2007; Schirmer et al., 2005; Di Ciaula et al., 2018, 2019; Gutt et al., 2020). The clinical presentation of cholecystolithiasis varied from the asymptomatic carrier status, to uncomplicated symptomatic disease with pain attacks, and complicated symptomatic disease, including acute cholecystitis, common bile duct stones, pancreatitis, cholangitis, and bowel obstruction(Shabanzadeh, 2018). In the current study, we have conducted the clinical, genetic, and molecular characterization of three Chinese families with cholecystolithiasis. Of these, 8 of 18 matrilineal relatives had a clinical phenotype, the penetrances of cholecystolithiasis were 75%, 33.3%, and 40%, respectively. Furthermore, the age at onset for cholecystolithiasis in those subjects varied from 31 to 43 years old, with an average of 38.7 years, slightly younger than the reported average age of onset(Chen et al., 2014). The majority of cholecystolithiasis patients show abnormal levels of cholesterol and lipoprotein in the blood (Shabanzadeh et al., 2016), but in our study, the cholesterol levels of all three pedigrees probands were normal while their HDL levels decreased significantly, especially inpatient A40.

Cholecystolithiasis is a multifactorial disease, the pathogenesis involves both genetic and environmental factors, of these, genetic factors account on an average for 25–29% of the risk (Nakeeb et al., 2002; Stokes et al., 2011; 2014). The nuclear gene *ABCB4* (ATP-binding cassette B4) is closely related to the occurrence of cholecystolithiasis (Jirsa et al., 2014; Elderman et al., 2015). In addition, the variants of *ABCG8* and *UGT1A1* have been identified to be involved, it may contribute to 21.2% the male population (Buch et al., 2010; Buch et al., 2010). Cholecystolithiasis are currently found in the maternal lineages of these families, suggesting that mtDNA mutations may be the molecular basis of this disease. The sequence analysis of the complete mitochondrial genomes in these pedigrees identified 625G > A mutation in the T-loop of the tRNA^phe^ gene. Indeed, Transfer RNAs (tRNAs), acting as adapters between nucleic acid and protein, plays an important central role in translation, and the secondary and tertiary structure of tRNAs are highly conserved (Shi and Moore, 2000; Machnicka et al., 2014). Mitochondrial tRNA mutations are thought to be associated with many multisystem disorders among the Chinese population. Of these, the two most common pathogenic mt-tRNA variants: m.3243A > G (within MT-TL1, encoding mt-tRNA^Leu(UUR)^) and m.8344A > G (within MT-TK, encoding mt-tRNA^Lys^), which account for the vast majority of all mt-tRNA-related diseases (Richteret al., 2021). In addition, mitochondrial tRNA mutations are involved in other isolated organ-specific diseases, such as deafness, tic disorders, Leber’s hereditary optic neuropathy, coronary heart disease, hypertension, etc., (Qin et al., 2014; Xue et al., 2016; Jiang et al., 2020; Zheng et al., 2020; Shuaiet al., 2021) The T-loop in tRNA, as a promoter for both the heavy and light strands of the mtDNA, is critical for tRNA structural stability (Anderson et al., 1981). The phenylalanine at position 49 in tRNA is extremely conserved among different organisms. This site mutation may lead to instability of the T-stem, which in turn leads to the destruction of the tRNA secondary structure. In addition to the 625G > A mutation, the pathogenic mitochondrial mutation site that has been reported is located in T-stem, such as 1662C > T, 4310A > G, 5558A > G, 5562U > A, 5772C > T, 5773C > T, 5783C > T, 5836T > C, 5846G > A, 10049A > T, 12254A > T, 14682G > T,14683T > C, 14696T > C, 15942T > C, 15944delT, 15941T > C, 15965T > C, and 15968A > G (http://www.mtdb.igp.uu.se/).

MitoTIP Sub-Scoring shows that m.625G > A mutation is more likely pathogenic. Indeed, the 625G > A mutation has been reported to be associated with deafness and epilepsy families (Sudo et al., 2011). In our study, the homoplasmic 625G > A mutation present only in the maternal lineage of those pedigrees but not other members of these families strongly indicates that this mutation may be associated with the pathogenesis of cholecystolithiasis. The varying degrees of penetrance of cholecystolithiasis indicated that the m.625G > A mutation, similar to other mitochondrial mutations (Liang et al., 2009; Liang et al., 2014), is itself insufficient to produce the clinical phenotype, the modifier factors including mitochondrial haplotypes are necessary for the phenotypic manifestation (zhao et al., 2009; Liang et al., 2014; Ji et al., 2014).

Mitochondrial haplotypes are defined by the combinations of single nuclear polymorphisms (SNPs) in mtDNA inherited from a common ancestor(Wallace. 2013). The tree of Native American mtDNA showed that almost all identified haplotypes clustered into only A, B, C, and D four distinct basal branches. After Native Americans, the Asian-specific haplogroups E, F, and G were identified in Tibetans, haplogroups H, I, J, and K in North Americans of European descent, haplogroups L, L1, and L2, the most ancient of all continent-specific haplogroups, were found in Sub-Saharan Africans, the Asian-specific haplogroup M was then identified, haplogroups T, U, V, W, and X were identified in Europeans (Antonio et al., 2020). Mitochondrial haplotype plays an important role in the phenotypic expression of diseases. Mitochondrial haplogroup G is associated with nonalcoholic fatty liver disease (Gusdonet al., 2021). The specific mtDNA background B5a1 was significantly associated with Southeast Asian G11778A LHON and appeared to modify the risk of visual loss (Kaewsutthi et al., 2011). Mitochondrial haplogroup J was associated with a higher risk of obesity in the Qatari population(Dashti et al., 2021). Mitochondrial DNA Haplogroup M7 Confers Disability in a Chinese Aging Population(Sun et al., 2020). Here, these mitochondrial genomes of A6, A7, and A40 pedigrees belong to the Eastern Asian haplogroups H2, F4b, and M10a, respectively. In the other studies, two Chinese cholecystolithiasis pedigrees carrying the m.827A > G belonged to the haplogroups B4a and B4c (Sun et al., 2021). This implied that the m.625G > A mutation, similar to other mitochondrial mutations, occurred sporadically and multiplied through the evolution of the mtDNA (Liang et al., 2014).

At present, the pathogenesis of cholecystolithiasis has not been well explained. The most commonly accepted hypothesis is gallbladder dyskinesia, caused by a pathological change in the gallbladder wall (Cafasso and Smith, 2014). However, the mechanism of gallbladder dyskinesia is not well understood. In fact, mitochondria provide a constant source of energy for human physiological activities, mitochondrial mutations can affect the ATP production. The m.625G > A mutation disturbed the tRNA secondary structure, and it may affect protein translation and thus energy production. Therefore, we speculate that the gallbladder sphincter muscle is unable to contract well due to a lack of ATP, resulting in gallbladder stones that cannot be discharged and cleared. On the other hand, mitochondrial dysfunction may also affect lipid metabolism. These may be the important reason for the occurrence of cholecystolithiasis. However, other genetic factors, other than 625G > A mutation, and environmental factors also play an important role in cholecystolithiasis disease.

## Data Availability

The original contributions presented in the study are publicly available.The datasets generated for this study can be found in the GSA for Human and the accession number is: HRA002404, https://bigd.big.ac.cn/gsa-human/browse/HRA002404.
